# Alcohol-induced epigenetic changes prevent fibrosis resolution after alcohol cessation in miceresolution

**DOI:** 10.1097/HEP.0000000000000675

**Published:** 2023-11-09

**Authors:** Michael Schonfeld, Maura O’Neil, Steven A. Weinman, Irina Tikhanovich

**Affiliations:** 1 Department of Internal Medicine, University of Kansas Medical Center, Kansas City, Kansas, USA; 2 Department of Pathology, University of Kansas Medical Center, Kansas City, Kansas, USA; 3 Kansas City VA Medical Center, Kansas City, Missouri, USA

## Abstract

**Background and Aims:**

Alcohol-associated liver disease is a major cause of alcohol-associated mortality. Recently, we identified hepatic demethylases lysine demethylase (KDM)5B and KDM5C as important epigenetic regulators of alcohol response in the liver. In this study, we aimed to investigate the role of KDM5 demethylases in alcohol-associated liver disease resolution.

**Approach and Results:**

We showed that alcohol-induced liver steatosis rapidly resolved after alcohol cessation. In contrast, fibrosis persisted in the liver for up to 8 weeks after the end of alcohol exposure. Defects in fibrosis resolution were in part due to alcohol-induced KDM5B and KDM5C-dependent epigenetic changes in hepatocytes. Using cell-type–specific knockout mice, we found that adeno-associated virus-mediated knockout of KDM5B and KDM5C demethylases in hepatocytes at the time of alcohol withdrawal promoted fibrosis resolution. Single-cell ATAC sequencing analysis showed that during alcohol-associated liver disease resolution epigenetic cell states largely reverted to control conditions. In addition, we found unique epigenetic cell states distinct from both control and alcohol states and identified associated transcriptional regulators, including liver X receptor (LXR) alpha (α). *In vitro* and *in vivo* analysis confirmed that knockout of KDM5B and KDM5C demethylases promoted LXRα activity, likely through regulation of oxysterol biosynthesis, and this activity was critical for the fibrosis resolution process. Reduced LXR activity by small molecule inhibitors prevented fibrosis resolution in KDM5-deficient mice.

**Conclusions:**

In summary, KDM5B and KDM5C demethylases prevent liver fibrosis resolution after alcohol cessation in part through suppression of LXR activity.

## Graphical abstract

**Figure:**
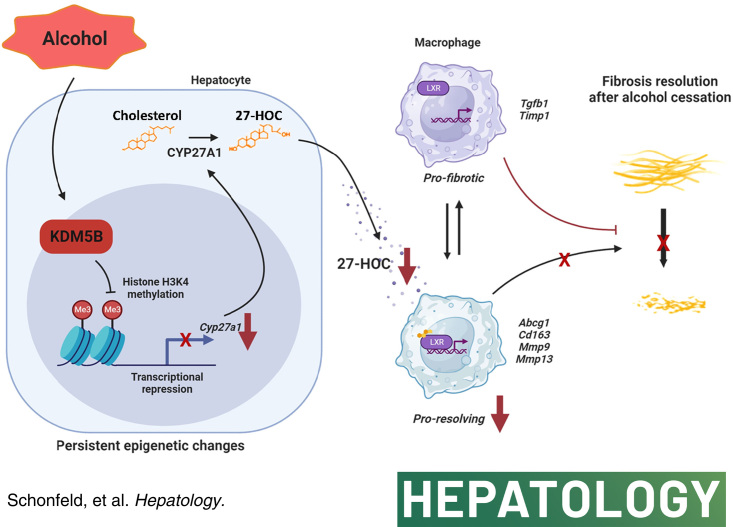

## INTRODUCTION

Alcohol-associated liver disease (ALD) encompasses a spectrum of disorders that commonly progresses from steatosis to steatohepatitis with fibrosis and ultimately to cirrhosis. Cirrhosis is the ninth leading cause of death in the United States, and about 35%–50% of cirrhosis deaths are alcohol-associated.^1,2^ In ALD, multiple liver cell types are impacted by alcohol exposure; however, it is not clear if the effects on individual cells are direct effects of alcohol or a response to alcohol-induced signals from other cells. Advances in technologies such as single-cell sequencing and spatial transcriptomics have revealed new details regarding the complexity of hepatic cell types^3–6^ and have highlighted the importance of cell-cell communication by means of secreted mediators such as peptides, hormones, and cytokines (secretome) in disease development and progression.^7^ Recent studies suggest that hepatocytes play a central role in driving ALD and influence the phenotype of non-parenchymal cells in the liver both during disease development^8,9^ and resolution.^10^

Abstinence is the most critical therapeutic intervention for patients with ALD. However, fibrosis improvement after drinking cessation is not uniform, and some patients progress to cirrhosis or develop decompensation even while abstinent.^1,11,12^ Fibrosis resolution after alcohol cessation is not well studied, mostly due to the absence, until recently, of animal models that can induce clinically relevant fibrosis. However, data from nonalcohol disease models suggest that even advanced stages of fibrosis can resolve when the cause of the liver injury is removed.^13^ This resolution involves apoptosis of activated HSCs or HSC reversal to a quiescent-like state,^14^ and collagen removal by increased matrix metalloproteinase (MMP)-dependent degradation^15–18^ and MRC-1-dependent uptake of degraded collagen by macrophages.^19^

KDM5B and KDM5C are enzymes that demethylate lysine 4 of histone H3 at active gene promoters, resulting in transcriptional repression.^20^ KDM5 demethylases do not have DNA binding specificity and rely on transcription factors to guide them to their target gene promoters.^21,22^ Thus, the functions of KDM5 demethylases are highly cell-type and context-dependent.^23^ *Kdm5b* KO mice display growth retardation, postnatal mortality, and altered insulin sensitivity. KDM5C is mostly known as a transcription repressor of neuronal genes, and it represses CLOCK-ARNTL/BMAL1-mediated transcription.^22^ Recently, we found that KDM5B and KDM5C are key regulators of alcohol responses in liver cells.^24,25^

In the current study, we have used the recently described Western diet with alcohol (WDA) mouse model^26^ that combines high-fat diet feeding and alcohol in the drinking water. This and similar high-fat/alcohol models have been used in multiple studies and have been shown to recapitulate multiple aspects of human ALD, including steatosis, inflammation, hepatocyte ballooning, and pericellular fibrosis.^26–28^ Using this model, we showed that the process of fibrosis resolution on alcohol cessation is extremely slow, and this lack of resolution is due to KDM5-dependent suppression of fibrosis resolution mechanisms. We found that hepatocyte-specific KO of *Kdm5b* or *Kdm5c* at the onset of alcohol cessation accelerated fibrosis resolution. This mechanism involved LXR activation in the liver with the subsequent development of pro-resolving changes in non-parenchymal cells. LXR inhibition by a small molecule inhibitor blocked fibrosis resolution in KO mice, suggesting that KDM5-mediated LXR suppression mediates poor fibrosis resolution after alcohol cessation.

## METHODS

### Mice

*Kdm5b* and *Kdm5c* floxed mice (B6/J^GptKdm5bem1C^ flox/wt and B6/J^GptKdm5cem1C^ flox/wt) were obtained from GemPharmatech Co., Ltd, Nanjing, China, and bred to make homozygous flox/flox breeders. All mice were housed in a temperature-controlled, specific pathogen-free environment with 12-hour light-dark cycles. All animal handling procedures were approved by the Institutional Animal Care and Use Committee at the University of Kansas Medical Center (Kansas City, KS).

In some experiments, liver fibrosis was induced by treating mice with 200 mg/L of thioacetamide (TAA) in the drinking water for 2 months.

### WDA model

For the WDA model as described,^26^ both male and female mice were fed ad libitum Western diet (WD) (Research Diets, Inc., Cat# D12079B), and alcohol was given ad libitum in water. Mice received progressively increasing amounts of alcohol in water (1%, 3%, 10%, 15%, and 20% for 3 days each). After reaching 20%, mice continued for 16–20 weeks. Alcohol-containing water was changed twice weekly.

### Vectors

AAV8-TBG-Cre, AAV8-CMV-Cre, and AAV8-TBG-EGFP were from Vector BioLabs, Malvern, PA; AAV8-CMV-Cre, and AAV8-CMV-EGFP were from Vector Builder, Chicago, IL and were used at 10^11^ genome copies per mouse each.

### Chromatin immunoprecipitation

Chromatin immunoprecipitation was performed as described.^29^ The detailed procedure is described in Supplemental Materials and Methods, http://links.lww.com/HEP/I95.

### Trans-well coculture

For coculture experiments, primary mouse hepatocytes were placed in cell inserts of 24-well trans-well plates (Corning Incorporated, Acton, MA, 0.4 µm pore size) at a seeding density of 5  ×  10^4^/well. Cells were treated as indicated. Freshly isolated liver macrophages were seeded in the bottom well at a seeding density 1  ×  10^4^/well. The cells were then cultured for 24 hours, and hepatocytes and macrophages were harvested for RNA isolation.

Alternatively, macrophages were used for a Collagen degradation assay, using DQ™ Collagen (ThermoFisher, Cat# D12060), type I From Bovine Skin, Fluorescein Conjugate, according to the manufacturer’s instructions. Cells were incubated in 50 mM Tris-HCl (pH 7.6), 150 mM NaCl, 5 mM CaCl2, 0.5% agar, 10 mg/L DQ™ Collagen for 2 hours at 37C. Digestion product fluorescence was measured at 525 nm.

### RNA-sequencing

For RNA-sequencing analysis, total RNA was isolated from the liver using the Qiagen RNA isolation kit. Three individual mice per condition were used. Library generation and sequencing were performed by BGI genomics services (BGI, Cambridge, MA). Twenty-seven samples were sequenced using the BGISEQ platform, on average generating about 4.57G Gb bases per sample. HISAT was used to align the clean reads to the reference genome. Bowtie2 was used to align the clean reads to the reference genes. The average mapping ratio with a reference genome (GRCm38.p6) was 96.14%, and 16869 genes were identified. Differential gene expression was identified with DESeq2. The data are available under GSE number GSE244240.

### Single-cell ATAC sequencing

Single-cell ATAC sequencing was performed by ActiveMotif Inc., Carlsbad, CA. Nuclei were harvested from liver tissue and subjected to library generation using 10x Genomics kit. 10x Genomics’ Cell Ranger ATAC analysis was performed on a per sample basis (BioProject ID: PRJNA1022784). Mapped reads were combined and analyzed using Signac and Seurat R packages as described.^3,30^ Trajectory analysis was performed using Monocle3 as described.^5,31^

### Statistics

Results are expressed as mean ± SD. The Student’s *t*-test, paired *t*-test, Pearson’s correlation, or one-way ANOVA with Bonferroni post hoc test was used for statistical analyses. *p*-value < 0.05 was considered significant.

### Data availability statement

Raw sequencing data for all reported datasets is available on request.

Additional methods are in the Supplemental Materials and Methods section, http://links.lww.com/HEP/I95.

## RESULTS

### ALD resolution is characterized by rapid steatosis reduction but no change in fibrosis

**FIGURE 1:**
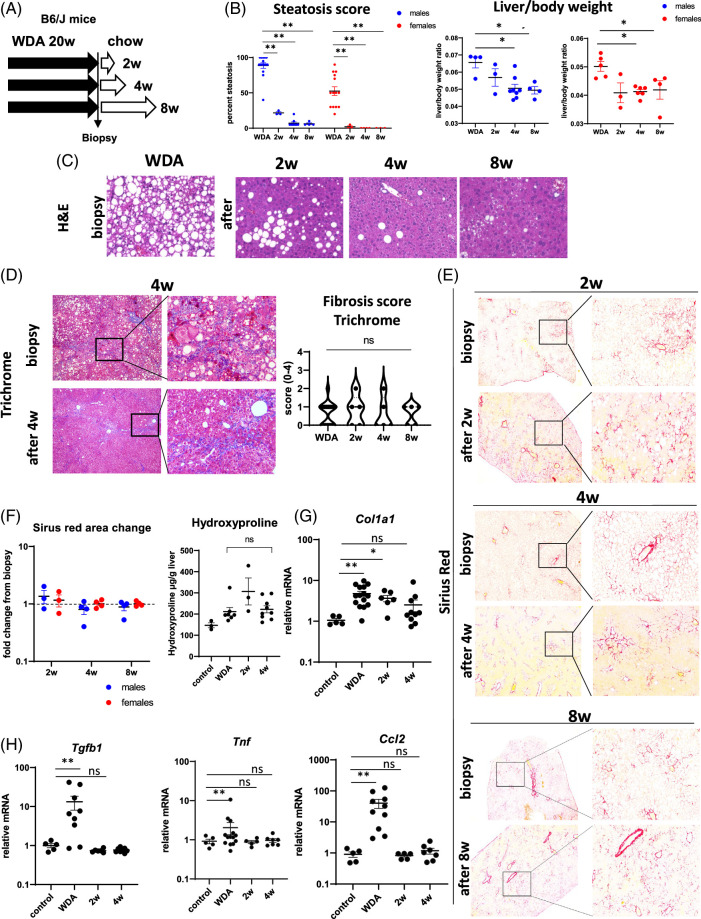
ALD resolution is characterized by rapid steatosis reduction but no change in fibrosis area. (A) Male and female 7–8 week old mice were fed ad libitum WDA in the drinking water for 20 weeks, then liver biopsy was collected, and mice were placed on a chow diet for 2–8 weeks. (B) Percent steatosis and liver/body weight ratios at indicated times. N ≥ 3 per group. *p < 0.05, **p < 0.01. (C) Representative images of H&E staining from biopsy (before) and livers after 2–8 weeks of resolution (after). (D) Corresponding biopsy and final liver sections were placed on the same slide and stained with Mason’s trichrome staining. The fibrosis stage was assessed and graded in a blinded fashion by a pathologist (Maura O’Neil). Fibrosis score and representative image of trichrome staining. (E) Corresponding biopsy and final liver sections were placed on the same slide and stained using Sirius red staining. Representative images from corresponding biopsy (before) and livers after 2–8 weeks of resolution (after). (F) Fold changes in Sirius red area from corresponding biopsy. Right. Hydroxyproline levels in the livers. (G–H) Relative mRNA expression in mice fed WD only (control), WDA (biopsy samples), and mice at 2–4 weeks of resolution. N ≥ 3 per group. *p < 0.05, **p < 0.01, ***p < 0.001. Abbreviations: WD, Western diet; WDA, Western diet with alcohol.

To assess disease resolution in mice fed alcohol, we used our recently described WDA model^26^ with slight modifications. In this model, mice consume ad libitum WD (43% calories from fat) and drink alcohol in drinking water (20% alcohol) for 20 weeks. At the end of alcohol feeding, we performed surgical liver biopsies and subsequently placed mice on a chow diet with plain water for 2–8 weeks (Figure 1A). We found that liver steatosis rapidly decreased (Figure 1B, C), and this correlated with a reduction in liver/body weight ratios. However, the pathological fibrosis score or the fibrosis area by Mason’s trichrome staining did not change (Figure 1D, Supplemental Figure S1, http://links.lww.com/HEP/I96). Next, we assessed the fibrosis area using Sirius red staining of liver sections at 2, 4, and 8 weeks and corresponding biopsy sections stained simultaneously on the same slide (Figure 1E, Supplemental Figure S2, http://links.lww.com/HEP/I97). We found that fibrosis area did not significantly change at any of the time points in both male and female mice (area fold change 0.98 ± 0.13 for males, 1.05 ± 0.07 for females), which correlated with no change in hydroxyproline levels in the liver (Figure 1F). Fibrosis resolution did not occur despite a decrease in pro-fibrotic and pro-inflammatory gene expression (Figure 1G, H). By 2 weeks after alcohol cessation, *Tgfb1, Tnf*, and *Ccl2* were decreased to the levels of control-fed mice (high-fat diet without alcohol) that do not develop liver fibrosis by Sirius red staining. By 4 weeks after alcohol cessation, *Col1a1* expression was also decreased back to control levels in the majority of mice. Taken together, these data suggest that fibrosis resolution is impaired in mice that stop alcohol after 20 weeks on the WDA diet.

### *Kdm5b* KO in hepatocytes promotes fibrosis resolution

**FIGURE 2:**
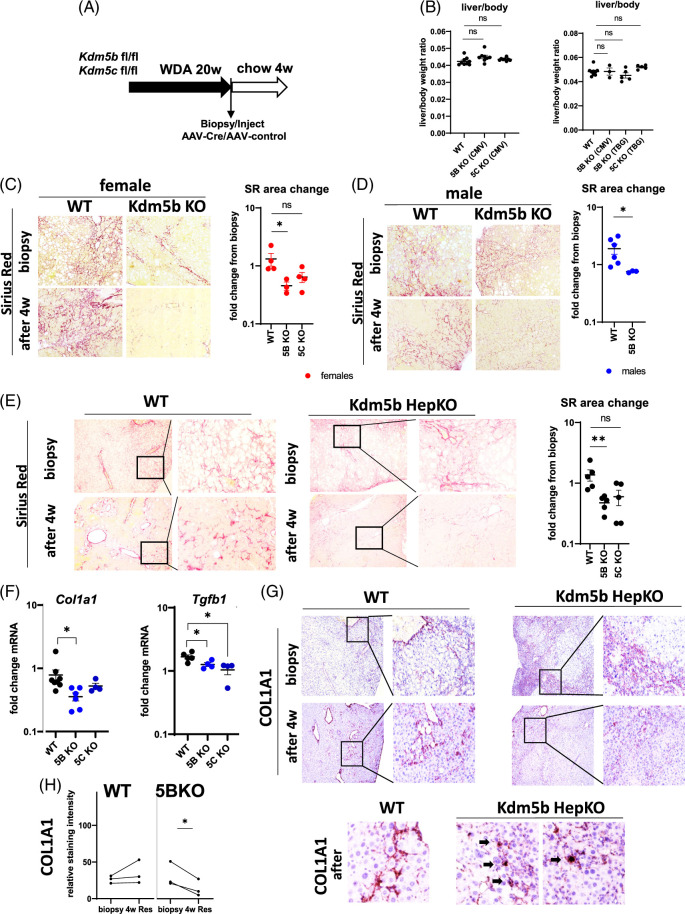
*Kdm5b* knockout promotes fibrosis resolution. (A) 7–8 week old *Kdm5b* fl/fl or *Kdm5c* fl/fl mice were fed ad libitum WDA in the drinking water for 20 weeks then liver biopsy was collected, and mice were placed on a chow diet. One day later, mice received 10^11^ gc/mouse of AAV-CMV-Cre or AAV-CMV-control, or AAV-TBG-Cre or AAV-TBG-control. (B) Liver/body weight ratios at 4 weeks of resolution. N ≥ 3 per group. (C–D) Mice were treated with AAV-CMV-Cre or AAV-CMV-control. Corresponding biopsy and final liver sections were placed on the same slide and stained using Sirius red staining. Representative images from corresponding biopsy (before) and livers after 4 weeks of resolution (after) in females (C) and males (D). Fold change in Sirius red area from the corresponding biopsy is presented on the right. (E) Mice were treated with AAV-TBG-Cre (HepKO) or AAV-TBG-control (WT). Representative images from corresponding biopsy (before) and livers after 4 weeks of resolution (after). N ≥ 3 per group. **p < 0.01. F. Relative mRNA expression in WT and KO (CMV-cre) mice at 4 weeks of resolution. N ≥ 3 per group. *p < 0.05. (G–H) Representative images from corresponding biopsy (before) and livers after 4 weeks of resolution (after) of COL1A1 staining in WT or hepatocyte-specific KO mice (TBG-Cre, HepKO). (H) Change in COL1A1 staining in WT and KO mice. N = 3, *p < 0.05. Right. Representative images of COL1A1 staining in zone 2. Arrows indicate fragmented collagen clusters. Abbreviations: AAV, adeno-associated virus; KDM, lysine demethylase; KO, knockout; WDA, Western diet with alcohol; WT, wild-type.

We have identified KDM5 demethylases as regulators of fibrosis development in female mice.^25^ To assess their role in post-alcohol fibrosis resolution, we used *Kdm5b* or *Kdm5c* floxed mice and induced gene KO at the time of alcohol cessation (Figure 2A, Supplemental Figure S3, http://links.lww.com/HEP/I98). Mice were then assessed for ALD resolution after 4 weeks. The KO did not affect steatosis resolution, and mice in all groups had comparable liver/body weight ratios and comparable histological steatosis resolution at the end of the experiment (Figure 2B, Supplemental Figure S4, http://links.lww.com/HEP/I99), but fibrosis resolution was enhanced in KO mice. Mice that received AAV-control showed no fibrosis resolution with Sirius red area fold change of 1.6 ± 0.4 in males and 1.3 ± 0.3 in females. Fibrosis levels at 4 weeks were slightly increased compared to experiments presented in Figure 1 likely due to AAV-control injections. We used 2 different AAV approaches to knock out *Kdm5b*. The first used AAV8-CMV.Cre, that targets the liver but is not cell-type–specific. These mice showed a significant reduction of fibrosis area (area fold change 0.7 ± 0.1 in males and 0.5 ± 0.1 in females, Figure 2 C, D, Supplemental Figure S5, http://links.lww.com/HEP/I100). In addition, a highly hepatocyte-specific KO using AAV8-TBG.Cre similarly reduced fibrosis area 4 weeks after alcohol cessation (area change 0.5 ± 0.1, Figure 2E). *Kdm5c* KO also promoted fibrosis resolution (Figure 2C, E, Supplemental Figure S5, http://links.lww.com/HEP/I100); however, this change was not significant (*p* > 0.05).

The increase in fibrosis resolution in KO mice correlated with reduced profibrotic gene expression in the liver (Figure 2F). Next, we examined collagen I in the livers of WT and KO mice using immunohistochemistry. We found that collagen staining did not change or slightly increased in WT mice after 4 weeks of resolution, while in KO mice, collagen staining and collagen content on immunoblot were reduced (Figure 2G, H, Supplemental Figure S6A, http://links.lww.com/HEP/I101). Moreover, we observed that in KO mice, collagen staining showed more fragmented collagen clusters in zone 2, which could be associated with increased degradation (Figure 2G).

### *Kdm5b* KO promotes pro-resolving gene expression in the liver

**FIGURE 3:**
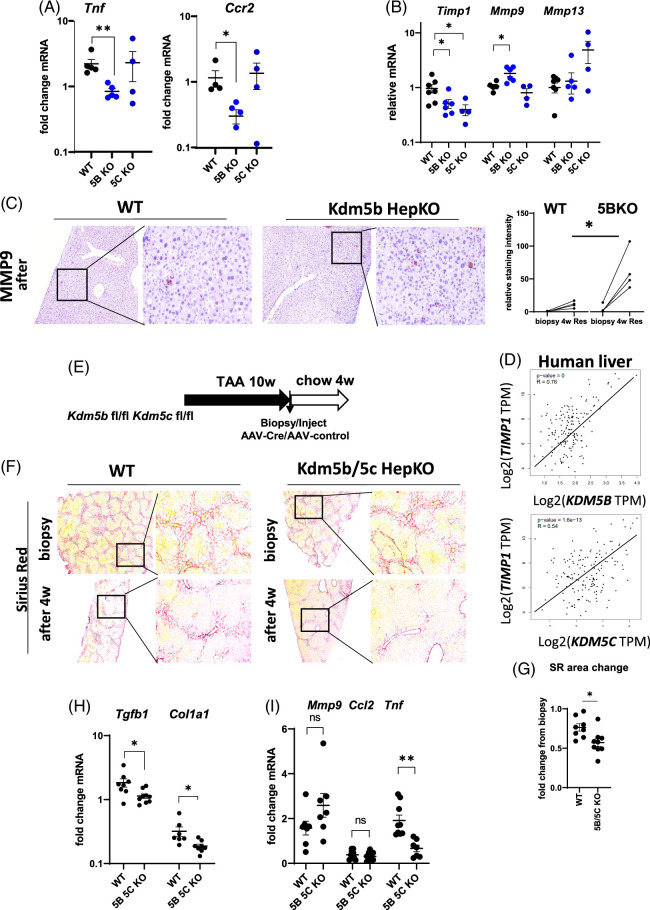
*Kdm5b* and *Kdm5c* knockout promotes pro-resolving gene expression in the liver. (A–B) Relative mRNA expression in WT and KO (CMV-cre) mice at 4 weeks of resolution, as in Figure 2. N ≥ 3 per group. *p < 0.05. (C) Representative images from corresponding biopsy (before) and livers after 4 weeks of resolution (after) of COL1A1 staining in WT or hepatocyte-specific KO mice (TBG-Cre, HepKO). (D) Correlation between *KDM5B* or *KDM5C* and *TIMP1* gene expression in human liver specimens from GTEx database. (E–I) 7-8 weeks old *Kdm5b* fl/fl *Kdm5c* fl/fl mice were fed ad libitum 200 mg/L of TAA in the drinking water for 10 weeks, then liver biopsy was collected, and mice were given plain water. One day after, mice received 10^11^ gc/mouse of AAV-TBG-Cre or AAV-TBG-control, and recovery was assessed after 4 weeks. (F) Corresponding biopsy and final liver sections were placed on the same slide and stained using Sirius red staining. Representative images from corresponding biopsy (before) and livers after 4 weeks of resolution (after) in WT (AAV-TBG-control) and *Kdm5b/Kdm5c* KO mice (HepKO, AAV-TBG-Cre). (G) Fold change in Sirius red area from corresponding biopsy N = 8–9 per group. *p < 0.05. (H–I) Relative mRNA expression (fold change from the biopsy) in WT and KO mice at 4 weeks of resolution. N ≥ 3 per group. *p < 0.05, **p < 0.01. Abbreviations: AAV, adeno-associated virus; KDM, lysine demethylase; KO, knockout; TAA, thioacetamide; WT, wild-type.

We have reported that KDM5B and KDM5C promote pro-inflammatory signaling in ALD.^25^ We examined pro-inflammatory gene expression changes in the livers of WT and KO mice during resolution (Figure 3A). We observed that in WT mice receiving AAV-control injections, inflammation was slightly increased at 4 weeks compared to the control (no AAV) experiment (Figure 3A compared with Figure 1). *Kdm5b* KO but not *Kdm5c* KO significantly reduced inflammation-associated gene expression (*Tnf, Ccr2*) in the liver at 4 weeks after alcohol cessation compared to WT mice. In addition, we observed that when compared to WT mice, alcohol cessation in both *Kdm5b* and *Kdm5c* KO mice was associated with an increase in matrix MMP gene expression and a decrease in expression of the MMP inhibitor, TIMP1 (Figure 3B). This suggests that the enhanced fibrosis resolution in the KO mice was associated with increased collagen degradation, possibly due to increased MMP activity. We further confirmed that the MMP9 protein level was increased in the livers of *Kdm5b* KO mice by immunohistochemistry and immunoblotting (Figure 3C, Supplemental Figure S6A) and further showed that increased MMP9 was specifically present in CD11b positive cells (Supplemental Figure S6B, http://links.lww.com/HEP/I101).

We assessed the relationship between KDM5 demethylases and *TIMP1* expression in human livers using a public database.^32^ We found that both *KDM5B* and *KDM5C* have strong positive correlations with *TIMP1* expression, suggesting that this mechanism is relevant in humans as well (Figure 3D).

Next, we investigated whether this mechanism is specific to ALD. We used 10 weeks of TAA treatment to induce liver fibrosis in *Kdm5b/Kdm5c* floxed mice, collected liver biopsies, then treated mice with AAV-TBG.cre or AAV-TBG.control and allowed them to recover for 4 weeks (Figure 3E). Unlike the ALD model, we found that TAA-treated WT mice showed a reduction in Sirius red staining after 4 weeks (Figure 3F, G). Nevertheless, *Kdm5b/Kdm5c* KO mice showed an even greater reduction in fibrosis area (fold change 0.6 ±0.1 in KO vs 0.8±0.1 in wild type). These changes correlated with reduced *Tgfb1, Col1a1*, and *Tnf* gene expression and increased *Mmp9* expression, although the difference in *Mmp9* mRNA did not reach statistical significance (Figure 3H, I).

### *Kdm5b* KO results in profound gene expression change during resolution that affect signaling from multiple nuclear receptors

**FIGURE 4:**
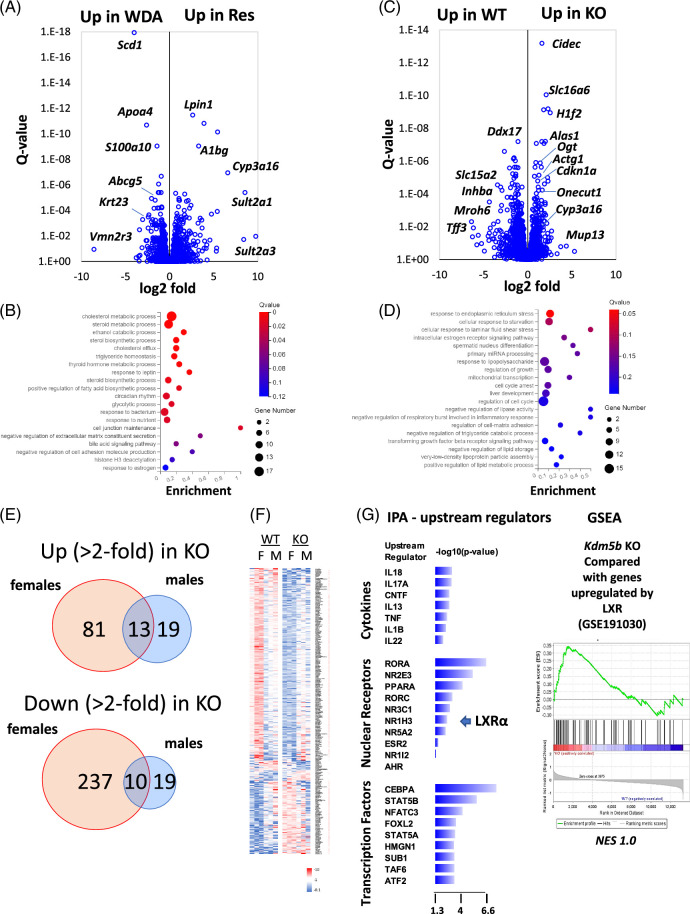
*Kdm5b* knockout promotes gene expression changes that affect multiple nuclear receptors signaling. (A) Volcano plot of differentially regulated genes in wild-type female mice fed WDA diet and WDA mice after 4 weeks of resolution (Res). N=3 mice per group. (B) Pathway enrichment in top differentially regulated genes during ALD resolution. (C) Volcano plot of differentially regulated genes in WT and *Kdm5b* KO(CMV-cre) female mice after 4 weeks of resolution. N = 3 mice per group. (D) Pathway enrichment in top differentially expressed genes in WT and KO mice. (E–F) Differentially regulated genes in WT and *Kdm5b* KO (CMV-cre) male and female mice after 4 weeks of resolution. N = 3 mice per group. (G) Ingenuity pathway analysis of upstream regulators in *Kdm5b* KO male and female mice (combined). Gene set enrichment analysis of gene expression changes in *Kdm5b* knockout male and female mice (combined) compared with gene set of the top 100 genes upregulated in WT mice livers compared to LXRα/β knockout mice livers. Abbreviations: ALD, alcohol-associated liver disease; KDM, lysine demethylase; KO knockout; LXRα, liver X receptor alpha; WD, Western diet; WDA, Western diet with alcohol; WT, wild-type.

To understand the mechanism of KDM5’s apparent ability to suppress fibrosis resolution after alcohol cessation, we performed RNA-sequencing analysis of whole liver mRNA from WT and *Kdm5b* KO mice fed alcohol and after 4 weeks of alcohol cessation. We found that during the resolution phase, there are profound changes in the liver transcriptome (Figure 4A), particularly in genes affecting metabolic pathways (cholesterol metabolism, glucose metabolism, bile acid metabolism), circadian rhythms, and extracellular matrix composition (Figure 4B). In *Kdm5b* KO mice compared to WT mice during resolution, most differentially regulated genes were involved in cell cycle, ER stress, TGFβ signaling, and lipid metabolism pathways (Figure 4C, D). Many genes that were differentially regulated in the KO mice had greater changes in females than in males (Figure 4E, F, Supplemental Figure S7, http://links.lww.com/HEP/I102). Unlike in our prior ALD development studies,^24,25^ we did not observe many genes that were affected by KO in males only. This suggests that the KDM5B roles in ALD development and resolution are different. To define the resolution-specific targets of KDM5B in males and females combined, we used the IPA upstream regulator analysis tool (Figure 4G). Transcriptional regulators that were predicted as targets of KDM5B included multiple nuclear receptors, such as LXRα, transcription factors, and cytokines, that are known to regulate fibrosis development in the liver. Using gene set enrichment analysis, we found that genes positively regulated by LXR (top 100 genes upregulated in WT livers compared to livers of LXRα/β KO mice, GSE191030) were enriched among genes upregulated in *Kdm5b* KO mice (Figure 4G), suggesting that LXR is one of the targets of KDM5B in the liver.

### Epigenetic changes in liver cells during ALD resolution

**FIGURE 5:**
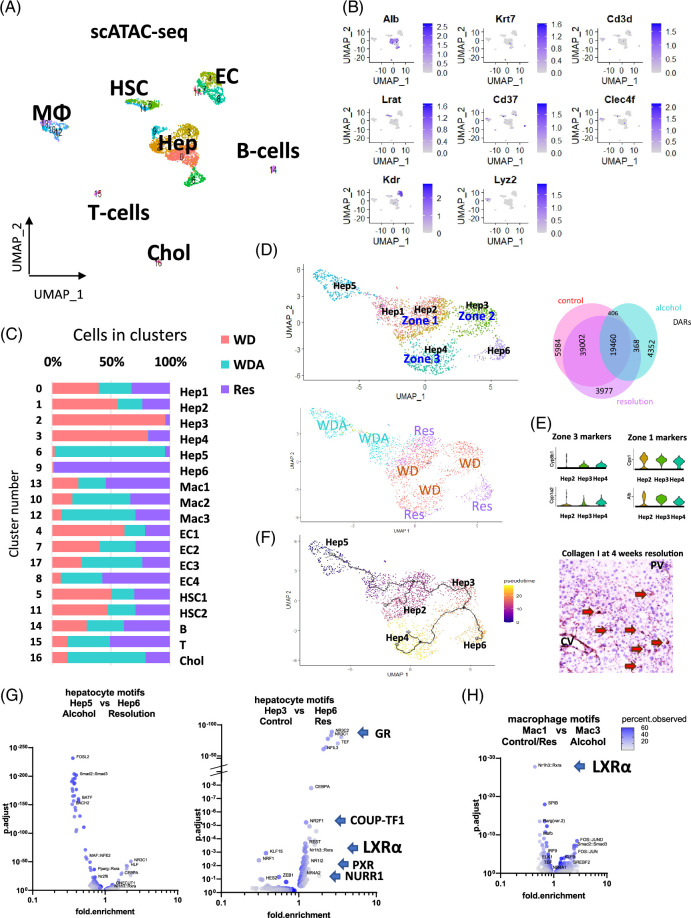
Epigenetic changes in liver cells during ALD resolution. Single-cell ATAC-seq analysis of liver cells from mice fed WD for 16 weeks (N = 1), WD with alcohol in the drinking water for 16 weeks (N=2, WDA), and 16 weeks of WDA followed by 4 weeks of resolution (Res, N=1). (A) UMAP clustering of liver cells. (B) Key cell type markers are used for cluster identification. (C) Relative abundance of cells from 3 conditions across clusters. (D) Hepatocytes re-clustered. Cells in hepatocyte clusters are colored according to their cluster (top panel) or the experimental conditions (bottom panel). A Venn diagram representing unique differentially accessible regions in the 3 conditions is shown on the right. (E) Zonation marker expression in clusters present in control (WD) condition. (F) Monocle3 trajectory analysis using WDA as a starting point. Zone 2 resolution cluster presents a separate branch of the trajectory. Right. Collagen I staining at 4 weeks of resolution in *Kdm5b* KO mice showing zone 2 collagen fragments indicated by arrows. (G–H) Motif enrichment analysis. Abbreviations: ALD, alcohol-associated liver disease; KDM, lysine demethylase; KO knockout; WD, Western diet; WDA, Western diet with alcohol.

To narrow down the list of potential regulators of ALD resolution, we explored epigenetic changes in the liver using single-cell ATAC sequencing analysis of livers from mice fed either WD, WDA, or WDA followed by 4 weeks of resolution. We identified 7 major cell types separated into 18 main clusters (Figure 5A, B). Among 6 hepatocyte clusters, Hep1 and Hep2 had cells from all three conditions. Hep3 was unique for WD cells, Hep5 for WDA, and Hep6 had cells only from WDA-resolution liver (Figure 5C, D). Differentially accessible regions overlapped greatly between the WD and WDA-resolution states (Figure 5D, right), while the WDA state differed greatly from both. We observed that hepatocytes in the WD and resolution conditions were separated based on liver zonation (Figure 5D, E); in zone 1 (Hep1 and Hep2) and in zone 3 (Hep4), resolution and control hepatocytes clustered together (Figure 5C, D), in zone 2 they formed distinct clusters (Hep3 and Hep6). Trajectory analysis using Monocle3^31^ confirmed that the unique resolution hepatocyte cluster (Hep 6) likely originates from zone 2 hepatocytes (Figure 5F). These data correlate with the observation of active collagen degradation in zone 2 in *Kdm5b* KO mice (Figure 5F, right), suggesting that hepatocyte-NPC crosstalk in zone 2 might regulate fibrosis resolution.

Next, we performed the analysis of motifs enriched in differentially accessible regions from different clusters (Figure 5G). We found that in hepatocytes from the WDA mice compared to WDA-resolution mice, one of the most enriched was the SMAD2/3 motif, which agrees with published data.^33^ In WDA-resolution hepatocytes, we found enrichment for several motifs, including LXRα (Figure 5G). In addition, the analysis predicted activation of the REST complex that includes H3K4 demethylases such as LSD-1 and KDM5 demethylases.^22^

We next compared differentially accessible regions in macrophage clusters Mac1 and Mac3. Mac1 was preferentially present in control and resolution conditions, and Mac3 in an alcohol-induced state (Figure 5C, H). Motif analysis identified LXRα as a top differentially activated regulator of Mac1 compared to Mac3 macrophages, suggesting that LXRα activity may play a role in multiple cell types during resolution.

### *Kdm5b* KO promotes LXR activation in liver cells

**FIGURE 6:**
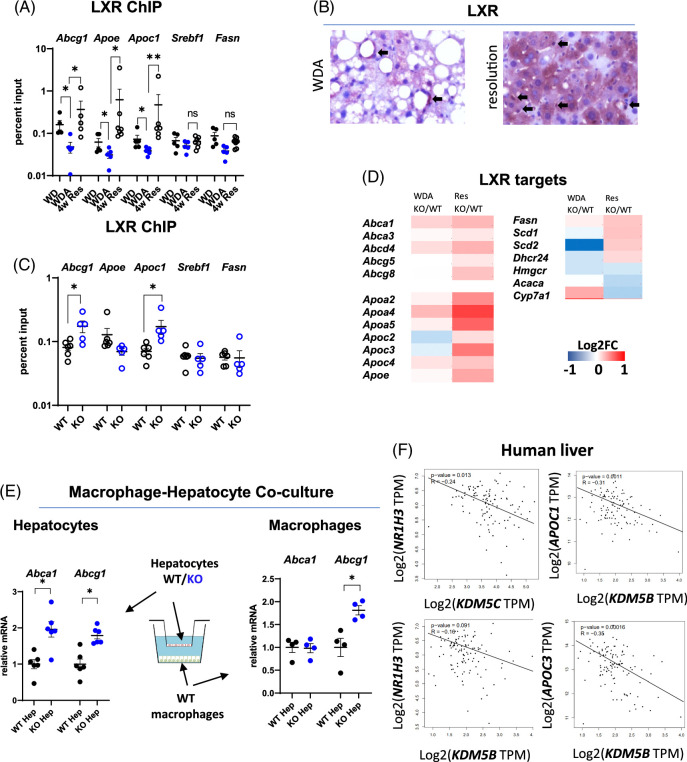
*Kdm5b* knockout promotes LXR activation in liver cells. (A) Chromatin immunoprecipitation assay using whole liver extracts from mice fed WD, WDA, or mice at 4 weeks of resolution. Data presented as percent input. N ≥ 4 per group. *p < 0.05, **p < 0.01. (B) LXRα/β protein staining in mice fed WDA diet or mice at 4 weeks of resolution. (C) Chromatin immunoprecipitation assay using whole liver extracts from WT or *Kdm5b* KO (CMV-cre) mice at 4 weeks of resolution. Data presented as percent input. N ≥ 4 per group. *p < 0.05, **p < 0.01. (D) Gene expression in *Kdm5b* KO (CMV-cre) mice relative to expression in WT mice fed either WDA or at 4 weeks of Res. (E) WT or *Kdm5b* KO hepatocytes were cocultured with WT liver macrophages in a trans-well coculture system. Relative mRNA in hepatocytes (left) and macrophages (right), N ≥ 4 per group. *p < 0.05. (F) Correlation between *KDM5B* or *KDM5C* and *NR1H3, APOC1*, *and APOC3* gene expression in human liver specimens from GTEx database. Abbreviations: KDM, lysine demethylase; LXRα, liver X receptor alpha; Res, resolution; WD, Western diet; WDA, Western diet with alcohol; WT, wild-type.

To assess the role of LXR during ALD development and resolution, we analyzed LXRα/β chromatin binding in the liver to a set of known target gene promoters (Figure 6A). We found that LXR binding to a subset of target genes, mostly involved in cholesterol efflux, was significantly reduced by alcohol. In contrast, binding to these genes was greatly enhanced during resolution (Figure 6A). This increase correlated with increased protein levels of LXRα/β (Figure 6B, Supplemental Figure S8A, http://links.lww.com/HEP/I103). In alcohol-fed mice, LXR was primarily expressed in F4/80 positive macrophages forming crownlike structures (Figure 6B, Supplemental Figure S8B, http://links.lww.com/HEP/I103). During resolution, LXR expression was elevated in both hepatocytes and macrophages throughout the liver parenchyma (Figure 6B, Supplemental Figure S8B, http://links.lww.com/HEP/I103). We found that *Kdm5b* KO during resolution further promoted LXR promoter binding to some of its targets (Figure 6C). Figure 6D shows RNA-sequencing data comparing KO to WT gene expression fold changes for both the WDA and resolution conditions. These data confirmed that expression of multiple LXR target genes is increased in KO mice specifically during the resolution phase (Figure 6D, Res). The increase was less pronounced in KO mice during the ALD development stage (Figure 6D, WDA).

We next tested if KDM5B regulated LXR target gene expression *in vitro*. We found that in mouse primary hepatocytes *Kdm5b* KO resulted in upregulation of LXR target genes, *Abca1* and *Abcg1*. In addition, when these hepatocytes were cocultured in a trans-well system with liver macrophages, the macrophage-specific LXR target gene (*Abcg1*) expression was increased in macrophages as well (Figure 6E), while gene expression of *Abca1*, not usually regulated by LXR in macrophages was unchanged. Finally, we assessed if this regulation is present in humans. We found that there was a significant negative correlation between *KDM5B* or *KDM5C* gene expression and LXRα (*NR1H3*) and its target genes *APOC1* and *APOC3* (Figure 6F).

### LXR promotes a pro-resolving phenotype in liver macrophages

**FIGURE 7:**
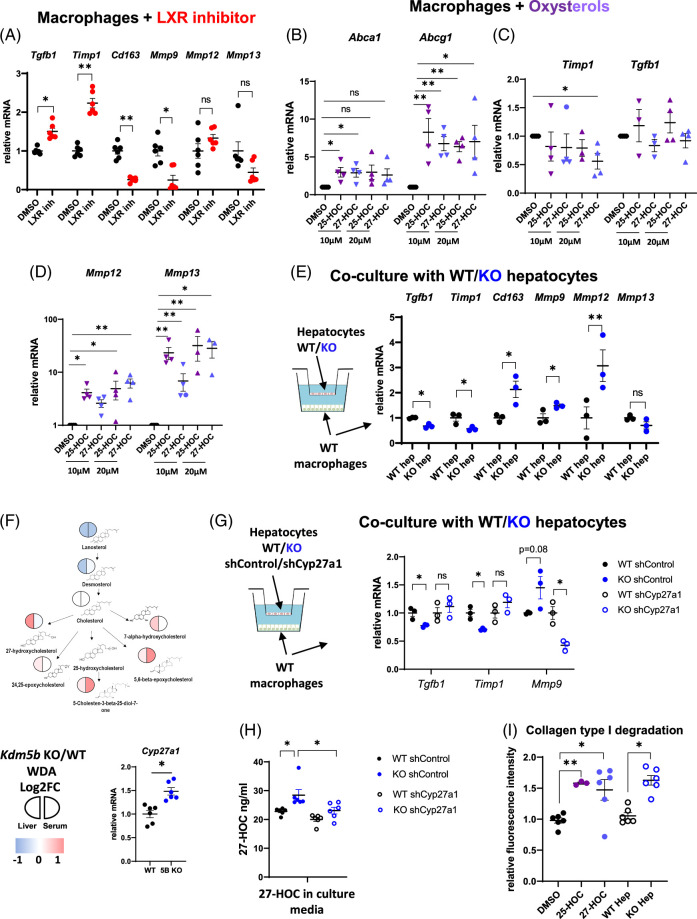
LXR promotes pro-resolving phenotype in liver macrophages. (A) Liver macrophages were treated with 1 µM of LXR inhibitor GSK2033 or DMSO control. Relative mRNA in macrophages, N ≥ 4 per group. *p < 0.05, **p < 0.01. (B–D) Liver macrophages were treated with 10 µM or 20 µM of 25-HOC or 27-HOC. Relative mRNA in macrophages, N ≥ 4 per group. *p < 0.05, **p < 0.01. (E) WT or *Kdm5b* KO hepatocytes were cocultured with WT liver macrophages in a trans-well coculture system. Relative mRNA in macrophages, N = 3 per group. *p < 0.05. (F) WT or *Kdm5b* KO mice were analyzed for metabolite concentrations in the liver and serum. Metabolite abundance relative to cholesterol. N = 3 per group. Right. *Cyp27a1* gene expression WT or *Kdm5b* KO hepatocytes 24 hours after induction of KO. (G–H) WT or *Kdm5b* KO hepatocytes were treated with shRNA specific to *Cyp27a1* or control shRNA and cocultured with WT liver macrophages in a trans-well coculture system. (G) Relative mRNA in macrophages, N = 3 per group. *p < 0.05. (H) 27-HOC levels in the culture media. N = 6 per group. *p < 0.05. (I) Relative collagen degradation activity in liver macrophages treated with 10 µM of 25-HOC or 27-HOC or conditioned media from WT or *Kdm5b* KO macrophages. Bottom, examples of DQ-Collagen-I signal in treated macrophage after 2 hours. Abbreviations: 25-HOC, 25-hydroxycholesterol; 27-HOC, 27-hydroxycholesterol; KDM, lysine demethylase; KO, knockout; WT, wild-type.

To evaluate the role of intrinsic macrophage LXR in macrophage pro-resolving and pro-fibrotic functions, we inhibited LXR activity using an LXR inhibitor (GSK2033) or activated LXR by adding oxysterols, 25-HOC or 27-HOC (Figure 7). LXR inhibitor treatment reduced *Cd163* expression, one of the top LXRα targets in liver macrophages.^34^ The inhibitor also induced pro-fibrotic gene expression (*Tgfb1* and *Timp1*) while reducing pro-resolving genes such as MMP genes (Figure 7A). In contrast, treatment with oxysterols induced the LXR target *Abcg1*, slightly reduced *Timp1* (Figure 7C) and increased MMP gene expression (Figure 7D). Overall, LXR activity positively correlated with a pro-resolving phenotype of macrophages. Consistent with our findings that KO hepatocytes promoted LXR activity in macrophages (Figure 6E), we observed that coculture of macrophages with KO hepatocytes reduced *Tgfb1* and *Timp1* and increased MMP gene expression (Figure 7E).

We hypothesized that LXR activation was mediated by increased levels of endogenous ligands of LXR produced by *Kdm5b* KO hepatocytes. Using metabolomic analysis, we confirmed that levels of several oxysterols were increased in the livers and serum of *Kdm5b* KO mice, while levels of desmosterol, another LXR agonist,^35^ were decreased (Figure 7F). These changes correlated with about a 2-fold increased expression of enzymes such as *Cyp27a1* and *Cyp3a16* as well as other *Cyp3a* family genes (Figure 4C), while enzymes involved in desmosterol level regulation, such as DHCR24, were not changed. We confirmed that *Kdm5b* KO in primary hepatocytes induces *Cyp27a1* expression (Figure 7F). These data correlated with reduced KDM5B binding to *Cyp27a1* promoter and increased H3K4me3 methylation at *Cyp27a1, Cyp3a16*, and *Cyp3a59* promoters in livers of mice deficient in *Kdm5b* (Supplemental Figure S9A, B, http://links.lww.com/HEP/I104) suggesting that they are direct targets of KDM5B.

Next, we studied the effect of *Cyp27a1* knockdown in hepatocytes on cell-cell crosstalk. We found that *Cyp27a1* knockdown abolished pro-resolving changes induced by *Kdm5b* KO in the macrophage-hepatocyte coculture system (Figure 7G). This correlated with reduced 27-HOC levels in the culture media (Figure 7H), suggesting that CYP27A1-mediated oxysterol production contributes to hepatocyte-NPC crosstalk.

We further tested whether pro-resolving gene expression changes correlated with increased collagen type I degradation using an *in vitro* collagen degradation assay. We observed that macrophages treated with oxysterols or exposed to KO hepatocyte-conditioned media had an increased ability to degrade type I collagen (Figure 7I), suggesting that *Kdm5b* KO in hepatocytes produces functionally important fibrosis resolution changes in macrophages.

### *Kdm5b* or *Kdm5c* KO in hepatocytes promotes fibrosis resolution through LXR activation

**FIGURE 8:**
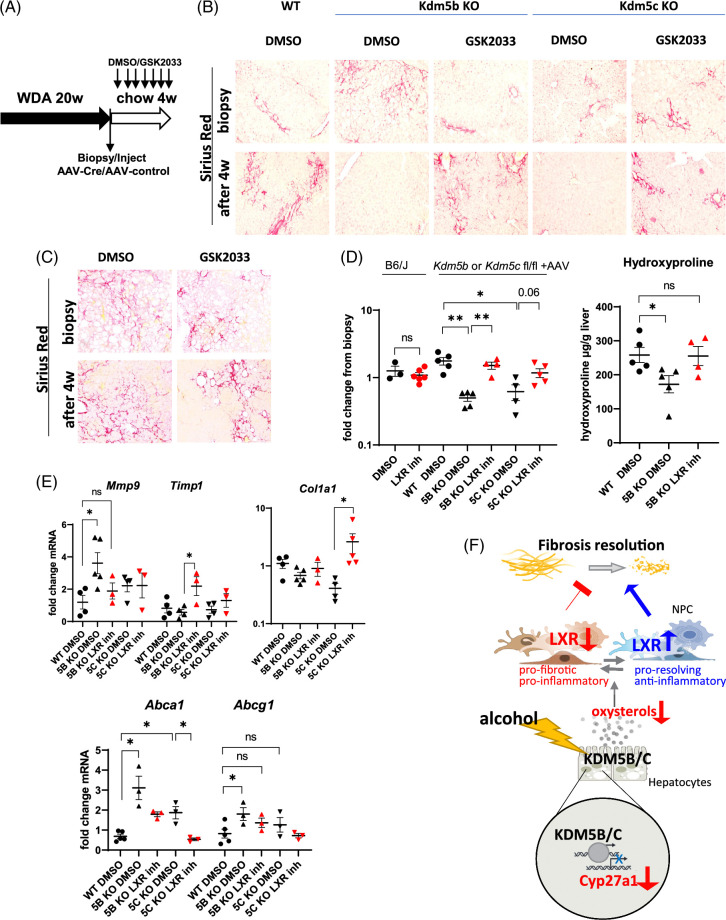
LXR activation is necessary for fibrosis resolution in *Kdm5b* or *Kdm5c* knockout mice. (A) 7–8-week-old B6/J, *Kdm5b* fl/fl or *Kdm5c* fl/fl mice were fed ad libitum WD and alcohol in the drinking water for 20 weeks, then liver biopsy was collected, and mice were placed on a chow diet. One day later, floxed mice received 10^11^ gc/mouse of AAV-CMV-Cre or AAV-CMV-control. Next, all mice were treated with 10 mg/kg of GSK2033 LXR inhibitor or DMSO control twice weekly for 4 weeks. (B–C) Corresponding biopsy and final liver sections were placed on the same slide and stained using Sirius red staining. Representative images from corresponding biopsy (before) and livers after 4 weeks of resolution (after). (D) Fold change in Sirius red area from the corresponding biopsy. Right. Hydroxyproline levels in the livers. N ≥ 3 per group. *p < 0.05, **p < 0.01. (E) Relative mRNA expression (fold change from the biopsy) in WT and KO mice at 4 weeks of resolution. N ≥3 per group. *p<0.05. (F) Model of KDM5-mediated cell-cell crosstalk in the liver during fibrosis resolution. Abbreviations: AAV, adeno-associated virus; KDM, lysine demethylase; KO, knockout; WD, Western diet; WT, wild-type.

To assess the interaction between LXR and KDM5 demethylases in fibrosis resolution, we treated WT and *Kdm5b* or *Kdm5c* KO mice with the LXR inhibitor during the resolution phase (Figure 8A). We found that LXR inhibition prevented fibrosis resolution in KO mice (Figure 8B), while it had no effect on the lack of resolution in WT controls (Figure 8C, D). LXR inhibition reduced *Mmp9* and increased *Timp1* gene expression in *Kdm5b* KO mice, while it promoted *Col1a1* and *Timp1* in *Kdm5c* KO mice, suggesting that some of the fibrosis resolution mechanisms are specific for either *Kdm5b* or *Kdm5c*. In both cases, LXR inhibition reduced the expression of LXR target genes in the liver (Figure 8E).

Taken together, these data suggest that KDM5B and KDM5C prevent fibrosis resolution after alcohol cessation in part through LXR inhibition and that LXR activity is necessary for efficient fibrosis resolution in KDM5 KO mice.

## DISCUSSION

Our data indicate that in ALD, fibrosis resolution on alcohol cessation is extremely slow, and this is different from the situation in other liver fibrosis models induced by chronic liver injury. Multiple studies of fibrosis resolution using the CCl_4_-fibrosis model have demonstrated a reduction in fibrosis area by 40%–60% after 4 weeks of resolution.^15,36^ We observed similar results using a TAA-fibrosis model (Figure 3). In ALD, however, even though collagen production is drastically reduced, there is no reduction in liver fibrosis for up to 8 weeks, suggesting that there is a defect in collagen degradation. One possible explanation is that alcohol induces changes in the liver that persist after alcohol cessation, for example, epigenetic changes, as evidenced by our ATAC-seq data, which prevent collagen degradation normally occurring during disease resolution. In this work, we identified the H3K4-specific demethylases KDM5B and KDM5C as mediators of alcohol-induced epigenetic changes that prevent collagen degradation and fibrosis resolution after alcohol exposure.

MMP-dependent collagen degradation is an important step in scar tissue degradation and fibrosis resolution. Several MMPs have been implicated in fibrosis resolution in the liver, including MMP2, MMP9, MMP13, and others.^15–18,37^ Interestingly, MMPs were also associated with fibrosis development in chronic injury models in mice^38^ and in humans with chronic liver disease.^16^ In our study, we observed that after alcohol cessation, MMP activity and MMP/TIMP ratio positively correlated with fibrosis resolution.

We found that the MMP/TIMP ratio was enhanced, and collagen production was decreased in mice lacking KDM5B and KDM5C in hepatocytes. These data suggest that hepatocytes are important players in fibrosis resolution. The role of hepatocytes in fibrosis resolution and development is not well studied. Many studies have focused on hepatocyte death as a trigger of fibrogenesis, and only a few studies have examined the hepatocyte-NPC communication pathways that contribute to pro-fibrotic versus pro-resolving phenotypes of other cells in the liver. Researchers have identified a number of hepatocyte-produced chemokines such as CCL-2 and CCL-5,^39^ HMGB-1,^40^ hepatocyte-derived Evs,^41^ and other factors that control ALD progression. However, the role of hepatocyte-produced factors in ALD fibrosis resolution is not clear. A recent study has reported the role of bile acids in post-injury fibrosis resolution in the liver.^36^ In our study, we found that hepatocyte-produced oxysterols intermediates in bile acid metabolism and mediate fibrosis resolution. Oxysterols are intermediates in the synthesis of bile acids, steroid hormones, vitamin D3, and other molecules. Their levels vary greatly in disease states.^42–44^ Oxysterols serve as agonists for the transcription factors LXRα and LXRβ in HSCs, LSECs, and liver macrophages,^42,43,45^ thus preventing excessive inflammation and HSC activation. Our data suggest that KDM5 demethylases regulate enzymes involved in oxysterol biosynthesis in the liver, such as *Cyp27a1* and the *Cyp3a* family, thus modulating LXR activation in liver cells.

LXRα and LXRβ are nuclear receptors that promote cholesterol efflux, gluconeogenesis, and lipogenesis. However, they are also known for their role in inflammation and liver fibrosis. Increased LXRα expression has been detected in quiescent HSCs,^46^ and it is a key transcription factor in Kupffer cells.^47^ LXR KO animals have been shown to develop higher levels of fibrosis.^48^ Some of the functions of LXR in liver fibrosis can be attributed to its role in the induction of cholesterol efflux genes since the accumulation of cholesterol in stellate cells and Kupffer cells aggravates liver fibrosis in mice.^44,49,50^ We found that at 4 weeks after alcohol cessation, *Kdm5b* KO mice had increased binding of LXR to promoters of its cholesterol efflux target genes *Abcg1* and *Apoc1*, but not *Srebf1* and *Fasn*. In coculture experiments, we found that *Kdm5b* KO hepatocytes promoted LXR target gene expression in hepatocytes and in macrophages, suggesting that hepatocyte KDM5 can modulate macrophage LXR activation. Using the LXR inhibitor GSK2033, we found that LXR activation generated a pro-resolving gene expression pattern in macrophages *in vitro* (by increasing the MMP/TIMP ratio)*; in vivo* experiments confirmed that LXR activity was necessary for fibrosis resolution in *Kdm5b* and *Kdm5c* deficient mice. Taken together, our data suggest that hepatocyte KDM5 regulates fibrosis resolution through a CYP27A/CYP3A-oxysterol-LXR-TIMP/MMP axis (Figure 8F). Selective KDM5 inhibitors or LXR activators could be a new promising therapy to promote fibrosis resolution after alcohol cessation in patients with ALD.

## Supplementary Material
