# Supplementary material for: Alcohol-induced epigenetic changes prevent fibrosis resolution after alcohol cessation in miceresolution
Source: Hepatology. 2023 Nov 9;80(1):119–35. doi: 10.1097/HEP.0000000000000675 (PMC11078890; doi:10.1097/HEP.0000000000000675)
Supplement: Supplementary file 1 [file hep-80-119-s001.docx]

**Supplemental Materials and methods**

**Biopsy**

Small liver biopsies were collected as previously described (1). Mice were anesthetized with isoflurane; a small incision was made on the upper right side of the abdomen and the liver exposed. One lobe of the liver was carefully lifted, and a small piece of liver (3-5 mm) was excised with scissors. The tissue was immediately placed in zinc-formalin and RNA-later for further processing. The gap in the liver was closed with an absorbable hemostatic gelatin sponge (Vetspon #96002). The incision was closed with 5-0 absorbable surgical suture (Redilene Redisorb Fast Pro #VF493-M) and 7mm wound-clips (Reflex 7 #203-1000). Mice were then injected with 1ml saline (SC) and 1.0 mg/kg SR Buprenorphine (SC). Mice were placed on a heating pad and monitored until fully awake from anesthesia; thereafter mice were monitored daily for the next seven days. The wound-clips were removed on day 8 after surgery.

**ChIP**

Chromatin immunoprecipitation was performed as described previously (2, 3). Liver cells (1 × 10^7^) were cross-linked by the addition of 1% formaldehyde for 10 minutes. Cells were subsequently lysed with 10mM Tris-HCl (pH 8.0), 10 mM NaCl, 3 mM MgCl_2_, 0.5% NP-40. Nuclei were collected by centrifugation, resuspended in [1% SDS, 5 mmol/L EDTA, 50 mmol/L Tris-HCl (pH 8.0)] and sonicated to generate chromatin to an average length of ~100 to 500 bp. Samples in 1% Triton X-100, 2 mM EDTA, 20 mM Tris–HCl of pH 8.1, 150 mM NaCl, were subsequently immunoprecipitated overnight at 4°C with 4 μg ChIP-grade antibody. 20 µl of magnetic beads (Dynabeads M-280, Invitrogen) were used to purify immunocomplexes. Following purification, cross-links were reverted by incubation at 65°C for 6 h. Samples were purified with the Qiagen DNA purification kit.

**Cell isolation**

Liver cells were isolated by a modification of the method described by Troutman et al (4). Mouse livers were digested by retrograde perfusion with liberase via the inferior vena cava. The dissociated cell mixture was placed into a 50 mL conical tube and centrifuged twice at 50 *g* for 2 min to pellet hepatocytes. The NPC-containing cell supernatant was further used to isolate macrophages. The cell suspension was pelleted by centrifugation (700 g, 10min, 4°C) and resuspended in PBS and OptiPrep (Sigma) to a final concentration of 17 %. Afterwards, 5ml of the indicated suspension was placed in a 15ml polystyrene conical centrifuge tube (BD Biosciences) and overlaid with 5ml of a 9% Optiprep solution followed by 2 ml PBS. After centrifugation at 1,400 g for 20min at 4°C with decreased acceleration and without breaks, the various cell-types were arranged according to their density. HSC were enriched in the upper cell layer whereas KC and LSEC were separated as a second layer of higher density. Cell fractions were collected separately by pipetting. The KC/LSEC fraction was pelleted, and macrophages were isolated with F4/80+MicroBeads (MiltenyiBiotec) according to the manufacturer’s instructions. Cells were applied onto LS magnetic-activated cell sorting (MACS) columns (MiltenyiBiotec), which were placed within the magnetic field of a MACS separator and washed 3 times with MACS buffer (MiltenyiBiotec). Cells were eluted and then seeded into culture dishes.

**Immunohistochemistry and immunofluorescence**

Liver tissue sections (5 μm thick) were prepared from formalin-fixed, paraffin-embedded samples. Immunostaining on formalin-fixed sections was performed by deparaffinization and rehydration followed by antigen retrieval by heating in a pressure cooker (121°C) for 5 minutes in 10 mM sodium citrate, pH 6.0 as described previously (5). Peroxidase activity was blocked by incubation in 3% hydrogen peroxide for 10 minutes. Sections were rinsed three times in PBS/PBS-T (0.1% Tween-20) and incubated in Dako Protein Block (Dako) at room temperature for 1 hour. After removal of blocking solution, slides were placed into a humidified chamber and incubated overnight with a primary antibody, diluted 1:300 in Dako Protein Block at 4°C. Antigen was detected using the SignalStain Boost IHC detection reagent (catalogue # 8114; Cell Signaling Technology, Beverly, MA), developed with diaminobenzidene (Dako, Carpinteria, CA), counterstained with hematoxylin (Sigma-Aldrich), and mounted.

For immunofluorescence after washing with PBST, coverslips were incubated with Alexa Flour -conjugated secondary antibody (1:300) in 0.1 µg/ml DAPI for 1 hour in the dark at room temperature. Coverslips were washed and mounted with FluorSave Reagent (Calbiochem. La Jolla, CA). Slides were analyzed in a Keyence BZ-X800 microscope (Keyence Corporation of America).

**Western Blotting**

Protein extracts (50 µg) were subjected to 10% SDS-polyacrylamide gel electrophoresis (SDS-PAGE), electrophoretically transferred to nitrocellulose membranes (Amersham Hybond ECL, GE Healthcare), and blocked in 3% BSA/PBS at RT for 1 hour. Primary antibodies were incubated overnight at manufacturer recommended concentrations. Immunoblots were detected with the ECL Plus Western Blotting Detection System (Amersham Biosciences, Piscataway, NJ) or using near-infrared fluorescence with the ODYSSEY Fc, Dual-Mode Imaging system (Li-COR).

**RT-PCR**

RNA was extracted from livers using the RNeasy Mini Kit (Qiagen). cDNA was generated using the RNA reverse transcription kit (Applied Biosystems, Cat.No 4368814). Quantitative real time RT-PCR was performed in a CFX96 Real time system (Bio-Rad) using specific sense and antisense primers combined with iQ SYBR Green Supermix (Bio-Rad) for 40 amplification cycles: 5 s at 95 °C, 10 s at 57 °C, 30 s at 72 °C. mRNA concentrations were calculated relative to *Actb*.

**Primers**

| RT-qPCR |  | ChIP |  |
| --- | --- | --- | --- |
| mActb fwd | ATGTCACGCACGATTTCCCT | mCyp27a1 F | CCGACCTCCAGGTAACCATC |
| mActb rvs | CGGGACCTGACAGACTACCT | mCyp27a1 R | GACAGTGGCGAGCTAAGTTCT |
| mTnf fwd | CTGAGACATAGGCACCGCC | mAbcg1 F | GCACACTAGCGCTAAGGGAA |
| mTnf rvs | CAGAAAGCATGATCCGCGAC | mAbcg1 R | AACCTCCCGAGGTAGCTTGA |
| mCol1a1 fwd | TGGCCAAGAAGACATCCCTG | mApoe F | TCCATCCCTGCGAAGTGGTA |
| mCol1a1 rvs | GGGTTTCCACGTCTCACCAT | mApoe R | CACAGTCCCCAAGTAACCCG |
| mMmp9 fwd | CCCTGGAACTCACACGACAT | mApoc1 F | CTCTGGGGCCAACTCGG |
| mMmp9 rvs | TCACACGCCAGAAGAATTTGC | mApoc1 R | CTCCTTTCAAGAAGCAAAAGGGT |
| mTimp1 fwd | GTAAGGCCTGTAGCTGTGCC | mFasn F | CGAAACCAATTGGACACCGAG |
| mTimp1 rvs | AGCCCTTATGACCAGGTCCG | mFasn R | CCGGCCCATCACCCTATTG |
| mAbca1 fwd | AGGACTAGACTCCAAGTTCTTCA | mSrebf1 F | CGATGTCGTTCAAAACCGCT |
| mAbca1 rvs | TGGACACCTTCTATGACAATTCTAC | mSrebf1 R | AGGCAGTCTCAACCCGCTA |
| mAbcg1 fwd | TGTCAGATACGGCTTTGAGGG |  |  |
| mAbcg1 rvs | GATGTCGCAGTGCAGGTCTT |  |  |
| mTgfb1 fwd | TACGTCAGACATTCGGGAAGC |  |  |
| mTgfb1 rvs | TTTAATCTCTGCAAGCGCAGC |  |  |
| mCcl2 fwd | ACCTGGATCGGAACCAAATGAG |  |  |
| mCcl2 rvs | GCTGAAGACCTTAGGGCAGAT |  |  |
| mCd163 fwd | GCTGAGGATGTCGGTGTGAT |  |  |
| mCd163 rvs | TCCTGAACATCTGGACACTCC |  |  |
| mMmp12 fwd | GTGGTACACTAGCCCATGCTT |  |  |
| mMmp12 rvs | TCCACGTTTCTGCCTCATCAA |  |  |
| mMmp13 fwd | ATGAAGACCCCAACCCTAAGC |  |  |
| mMmp13 rvs | ATGGCATCAAGGGATAGGGC |  |  |
| mCyp27a1 fwd | GATCAGTGGAAGGACCACCG |  |  |
| mCyp27a1 rvs | CCATTGCTCTCCTTGTGCGA |  |  |

**Hydroxyproline assay**

Hydroxyproline assays were performed using a hydroxyproline assay kit (Cell Biolabs Cat# STA-675) according to manufacturer’s instructions.

**27-hydroxycholesterol ELISA**

27-hydroxycholesterol was detected in culture media using a 27-Hydroxycholesterol (27-HC) competitive ELISA Kit (Abbrexa, cat# abx257403) according to manufacturer’s instructions.

**Metabolomics**

Cholesterol Metabolism (Total Sterol lipids) Analysis was performed using UPLC-MS/MS by Creative Proteomics (Creative Proteomics, Shirley, NY). Briefly, the LC-MS platform consisted of a Shimadzu Prominence HPLC coupled to a Thermo LTQ-Orbitrap Velos mass spectrometer. The LC system included two LC20AD pumps, a vacuum degassing system, autosampler, and column oven. The HPLC column was a Phenomenex 2.0 mmx150 mm Synergi HydroRP-C18 (4 micron, 80Angstrom pore size) equipped with a guard cartridge of the same column chemistry. Solvent A was water containing 0.1% formic acid. Solvent B was methanol containing 0.1% formic acid. The flow rate was 250 microliters per minute and the column oven was held at 50 degrees C. The autosampler was held at 15 degrees C. 10 microliters of each sample was injected. The gradient conditions used were: Time 0-2 minutes, 85% solvent B. Column eluant was diverted to waste using a 2-position 6 port valve. Solvent B was increased linearly to 100% between 2.0 and 16 minutes. Solvent B was then held at 100% for 9 minutes. Solvent B was returned to 85% to re-equilibrate the column. Column eluent was introduced to a Thermo LTQ-Orbitrap Velos mass spectrometer via a heated electrospray ionization source. The mass spectrometer was operated in positive ion mode at 60,000 resolution with full scan MS data collected from 300-700 m/z. Data-depended product ion spectra were collected on the 4 most abundant ions at 30,000 resolution using the FT analyzer. The electrospray ionization source was maintained at a spray voltage of 4.5 kV with sheath gas at 30 (arbitrary units) and auxillary gas at 10 (arbitrary units). The inlet of the mass spectrometer was held at 350 degrees C, and the S-lens was set to 50%. The heated ESI source was maintained at 300 degrees Celsius. Sterol and oxysterol species were identified as their [M-H2O+H]+, [M-2H2O+H]+, [M+H]+, [M+Na]+ and [M+K]+ ions under the conditions employed. Prior to analysis of serum cholesterol, samples were diluted 50-fold in methanol. For analysis of non-cholesterol sterols, samples were analyzed undiluted. Chromatographic alignment, isotope correction, peak identification and peak area calculations were performed using MAVEN software. Concentrations of each analyte were determined against the peak area of the internal standard (D6 cholesterol for cholesterol, and 19-hydroxycholesterol for other sterols). Sterols are reported as the sum of each ion type identified (e.g. [M-H2O+H]+, [M-2H2O+H]+, [M+H]+, [M+Na]+ and [M+K]+ ions).

**Supplemental Figure legends**

**Figure S1** ALD resolution is characterized by rapid steatosis reduction but no change in fibrosis area. Male and female 7–8-week-old mice were fed ad libitum Western diet and alcohol in the drinking water for 20 weeks, then liver biopsy was collected, and mice were placed on chow diet for 2-8 weeks. Corresponding biopsy and final liver sections were placed on the same slide and stained with Mason’s trichrome staining. Examples of individual mice biopsies and liver sections at the time of sacrifice.

**Figure S2.** Male and female 7–8-week-old mice were fed ad libitum Western diet and alcohol in the drinking water for 20 weeks then liver biopsies were collected, and mice were placed on chow diet for 2-8 weeks. Corresponding biopsy and final liver sections were placed on the same slide and stained using Sirius Red staining. Examples of individual mice biopsies and liver sections at the time of sacrifice.

**Figure S3.** 7–8-week-old *Kdm5b* fl/fl or *Kdm5c* fl/fl mice were fed ad libitum Western diet and alcohol in the drinking water for 20 weeks. Mice received 10^11^ gc/mouse of AAV-CMV-Cre or AAV-CMV-control at the beginning of alcohol feeding. Alternatively, mice were treated with AAV and were placed on chow diet for 4 weeks. Representative images of immunohistochemistry staining using KDM5B or KDM5C specific antibodies are presented as indicated.

**Figure S4.** 7–8-week-old *Kdm5b* fl/fl or *Kdm5c* fl/fl mice were fed ad libitum Western diet and alcohol in the drinking water for 20 weeks then liver biopsies were collected, and mice were placed on chow diet. One day after mice received 10^11^ gc/mouse of AAV-CMV-Cre or AAV-CMV-control. Examples are shown of individual mice biopsies (top row) and liver sections at the time of sacrifice (bottom row) stained with H&E.

**Figure S5.** Mice were treated as in figure 2. Examples of individual mice biopsies and liver sections at the time of sacrifice.

**Figure S6.** Mice were treated as in figure 2. **A.** Wild type and *Kdm5b* KO (AAV-CMV-Cre) mouse livers were analyzed by western blotting using indicated antibodies. Ponceau staining used as loading control. **B.** Immunofluorescence staining using MMP9 specific and CD11b specific antibodies in liver section from *Kdm5b* KO mice (AAV-TBG-Cre). Arrows show co-localization.

**Figure S7**. Top up and down regulated genes in *Kdm5b* knockout mice (AAV-CMV-cre).

**Figure S8. A.** LXRα/β western blot analysis in mice at the end of WDA diet feeding (WDA) or 4 weeks after cessation of WDA (resolution). **B**. Immunofluorescence staining using LXRα/β specific and F4/80 specific antibodies.

**Figure S9.** Mice were fed WD diet (control) or WDA diet (alcohol) and treated with AAV-shControl or AAV-shKdm5b and AAV-shKdm5c. **A.** Chromatin immunoprecipitation assay from whole liver extracts using KDM5B specific antibodies. Data presented as percent input. N ≥3 per group. *, P<0.05, **, P<0.01. **B.** ChIP-seq analysis showing H3K4me3 peaks at indicated genome positions.

**Supplemental References**

1. Schonfeld M, Villar MT, Artigues A, Weinman SA, Tikhanovich I. Arginine Methylation of Integrin Alpha-4 Prevents Fibrosis Development in Alcohol-Associated Liver Disease. Cell Mol Gastroenterol Hepatol 2022;15:39-59.

2. Li Z, Zhao J, Tikhanovich I, Kuravi S, Helzberg J, Dorko K, Roberts B, et al. Serine 574 phosphorylation alters transcriptional programming of FOXO3 by selectively enhancing apoptotic gene expression. Cell Death Differ 2016;23:583-595.

3. Tikhanovich I, Zhao J, Olson J, Adams A, Taylor R, Bridges B, Marshall L, et al. Protein Arginine Methyltransferase 1 modulates innate immune responses through regulation of peroxisome proliferator-activated receptor gamma-dependent macrophage differentiation. J Biol Chem 2017.

4. Troutman TD, Bennett H, Sakai M, Seidman JS, Heinz S, Glass CK. Purification of mouse hepatic non-parenchymal cells or nuclei for use in ChIP-seq and other next-generation sequencing approaches. STAR Protoc 2021;2:100363.

5. Zhao J, Adams A, Roberts B, O'Neil M, Vittal A, Schmitt T, Kumer S, et al. PRMT1 and JMJD6 dependent arginine methylation regulate HNF4alpha expression and hepatocyte proliferation. Hepatology 2017.
